# The molecular mechanisms, roles, and potential applications of PANoptosis in cancer treatment

**DOI:** 10.3389/fimmu.2025.1550800

**Published:** 2025-04-29

**Authors:** Wenyuan Ma, Qiang Wang, Lanfang Guo, Xiaoli Ju

**Affiliations:** ^1^ Department of Pathology, The People’s Hospital of Danyang, Affiliated Danyang Hospital of Nantong University, Danyang Clinical Medical College of Jiangsu University, Danyang, Jiangsu, China; ^2^ School of Life Sciences, Jiangsu University, Zhenjiang, Jiangsu, China; ^3^ Department of Clinical Laboratory Medicine, The Fourth People’s Hospital of Jiangsu University, Zhenjiang, Zhenjiang, Jiangsu, China; ^4^ Department of Pathology, School of Medicine, Jiangsu University, Zhenjiang, Jiangsu, China

**Keywords:** PANoptosis, cancer therapy, PANoptosome, molecular mechanisms, tumor microenvironment

## Abstract

PANoptosis, a newly identified form of programmed cell death regulated by the panoptosome complex, exhibits key characteristics of apoptosis, pyroptosis and necroptosis. It exerts a substantial influence on the initiation and progression of a spectrum of diseases, particularly in cancer, where its impact is increasingly being recognized. PANoptosis is closely related to tumorigenesis, carcinogenesis, metastasis, chemotherapy resistance, as well as the prediction of therapeutic responses and prognosis in cancer patients. In this review, we first review the discovery of PANoptosis and systematically analyze the composition of the panoptosome. Subsequently, we examine the role of PANoptosis in various types of cancer, encompassing its function within the tumor microenvironment, its role in tumor drug resistance, and its predictive role in cancer prognosis. Ultimately, we delve into strategies for targeting PANoptosis in cancer therapy, including targeting various molecules in the PANoptosis pathway, such as ZBP1, RIPK1, RIPK3, Caspases and other novel strategies like nanoinducers and viral vectors. This review aims to provide references and assistance for the research and application of PANoptosis in cancer treatment.

## 1 Introduction

Inducing tumor cell death is an important strategy in cancer treatment. Currently, methods to induce tumor cell death mainly include chemotherapy, radiotherapy, immunotherapy and targeted therapy. Many of these approaches suppress tumor growth by inducing different types of cell death in tumor cells, ultimately achieving the goal of therapy (1).

These treatment methods can inhibit tumor growth by inducing both non-programmed cell death or programmed cell death (PCD), with a predominant focus on inducing PCD in tumor cells. The types of PCD that can be induced in tumor cells include: apoptosis, necroptosis, pyroptosis, ferroptosis, immunogenic cell death (ICD) and autophagy-dependent cell death (2). In recent years, researchers have identified new types of cell death, such as cuproptosis and disulfidptosis, as well as PANoptosis (3). These newly discovered forms of PCD have provided new perspectives and potential therapeutic targets for cancer treatment.

However, tumor cells are characterized by heterogeneity, evasion of PCD and resistance to specific types of cell death. Inducing certain types of PCD in tumor cells does not achieve the desired therapeutic effect (4). This requires the simultaneous regulation of multiple cell death signaling pathways or the discovery of more types of PCD to avoid the development of drug resistance.

The concept of PANoptosis was first proposed by Malireddi et al. in 2019 (5). It is a newly discovered type of inflammatory cell death regulated by the PANoptosome (6). This form of cell death possesses the main characteristics of the three types of programmed cell death: pyroptosis, apoptosis, and necroptosis. In recent years, PANoptosis has been found to be involved in the occurrence and development of various diseases, such as infectious diseases, cancer, neurodegenerative diseases and autoimmune diseases (7). In particular, the role of PANoptosis in cancer treatment is multifaceted, including suppressing tumor growth, enhancing chemotherapy sensitivity, predicting treatment response and prognosis, and promoting immune surveillance.

This review will systematically review the PANoptosis, its components and its various roles in cancer. Finally, the challenges and future directions of various strategies of PANoptosis for cancer treatment are analyzed.

## 2 The discovery of PANoptosis

**Figure 1:**
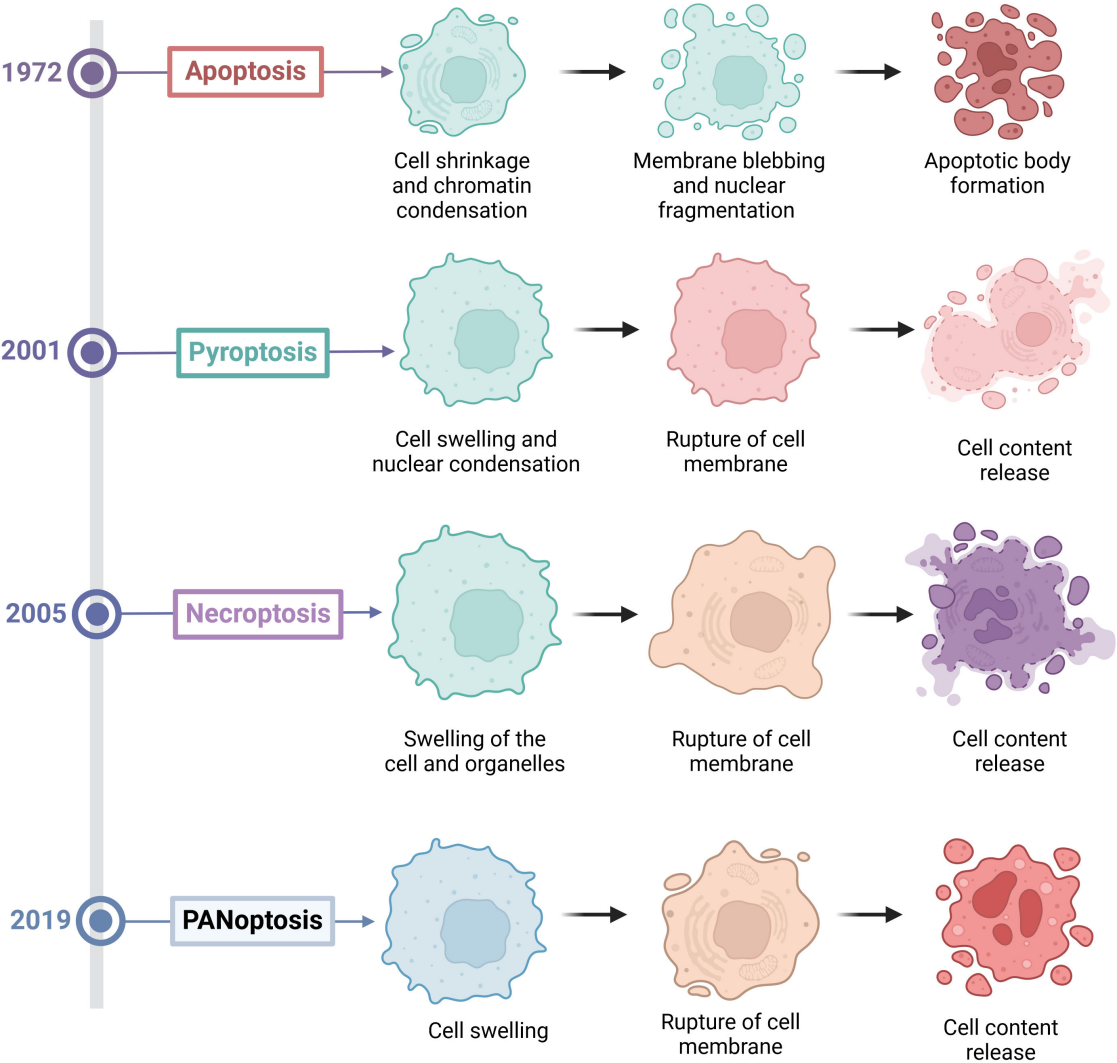
The discovery time and morphological characteristics of various forms of programmed cell death.

**Figure 2:**
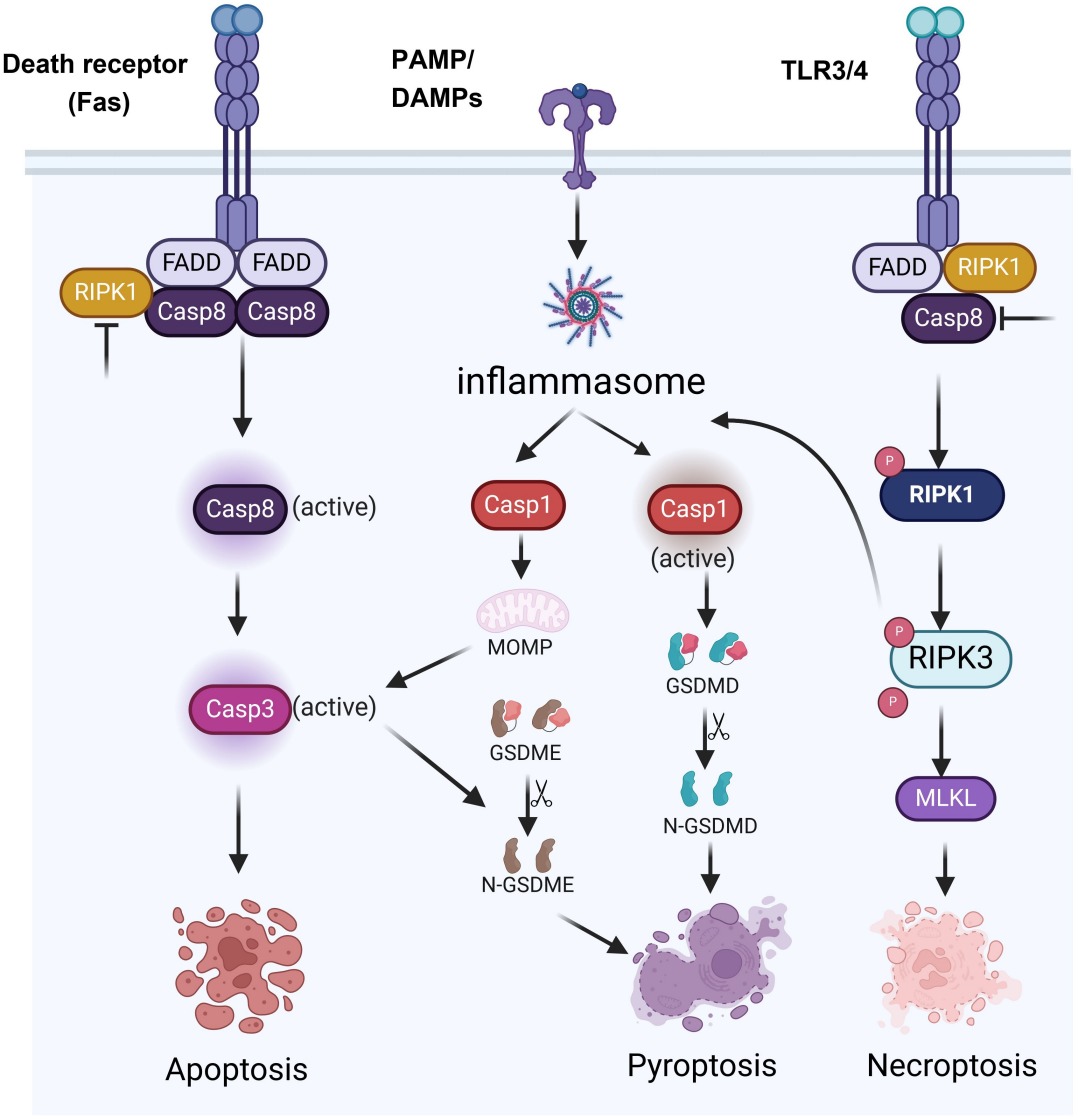
Signaling pathways of various forms of programmed cell death.

PCD is an important process of cell life. It plays a key role in maintaining homeostasis, the development of organisms, and disease defense. Among them, apoptosis, pyroptosis, and necroptosis are the forms of PCD that are most extensively studied (8). The three types of PCD have distinct morphological characteristics, molecular mechanisms and biological functions. Apoptosis is primarily characterized by nuclear condensation, membrane blebbing, cytoplasmic shrinkage and DNA fragmentation, and it typically does not cause inflammation (**Figure 1**). Apoptosis mainly depends on the activation of Caspases enzymes such as Caspase-3 (CASP3) and Caspase-8 (CASP8), as well as the regulation of Bcl-2 family proteins (9) (**Figure 2**). Pyroptosis and necroptosis are inflammatory cell death. Pyroptosis mainly depends on the Gasdermin proteins such as GSDMD and GSDME, which are mediated by inflammatory Caspases such as Caspase-1 (CASP1), Caspase-4 (CASP4), CASP3, and Caspase-11 (CASP11). Eventually, it leads to the formation of pores on the cell membrane, releasing cellular contents and triggering inflammatory reactions (10) (**Figure 2**). Necroptosis is characterized by cell swelling, membrane rupture, and the release of cellular contents (11) (**Figure 1**). It does not depend on Caspases activity but forms necroptosome and mediates the rupture of the cell membrane through the protein MLKL (**Figure 2**). Previous studies have indicated that these three types of cell death are independent and not associated with each other. However, recent biochemical, molecular, and genetic findings have shown significant crosstalk between these three types of PCD (12).

Kanneganti’group first discovered in 2016 that the influenza A virus (IAV) can be recognized by Z-DNA binding protein 1 (ZBP1) after infection (13). Subsequently, ZBP1 triggers apoptosis, necroptosis and pyroptosis in infected cells by regulating the activation of the NLRP3 inflammasome and through the RIPK1-RIPK3-CASP8 axis (27917412). In 2017, they further discovered that upon sensing the viral ribonucleoprotein (vRNP) complex of IAV, ZBP1 induces its own ubiquitination, leading to the induction of programmed cell death in infected cells (14). Finally, in 2019, they named this new form of cell death “PANoptosis,” where the letters P, A, and N represent pyroptosis, apoptosis, and necroptosis, respectively, and “optosis” represents a form of programmed cell death (5). PANoptosis is also an inflammatory form of PCD, which is activated by various triggers and regulated by PANoptosome. PANoptosis has the key characteristics of apoptosis, pyroptosis, and necroptosis, but cannot be simply explained by any one of these three PCD pathways alone (**Figure 1**). Moreover, blocking any one of these three death pathways is not effective in preventing the progression of PANoptosis. However, current methods for detecting PANoptosis (such as LDH assays and microscopy) cannot reliably distinguish PANoptosis from concurrent pyroptosis, apoptosis, or necrosis. A combination of multiple approaches is required for comprehensive detection.

## 3 Composition of the PANoptosome

**Figure 3:**
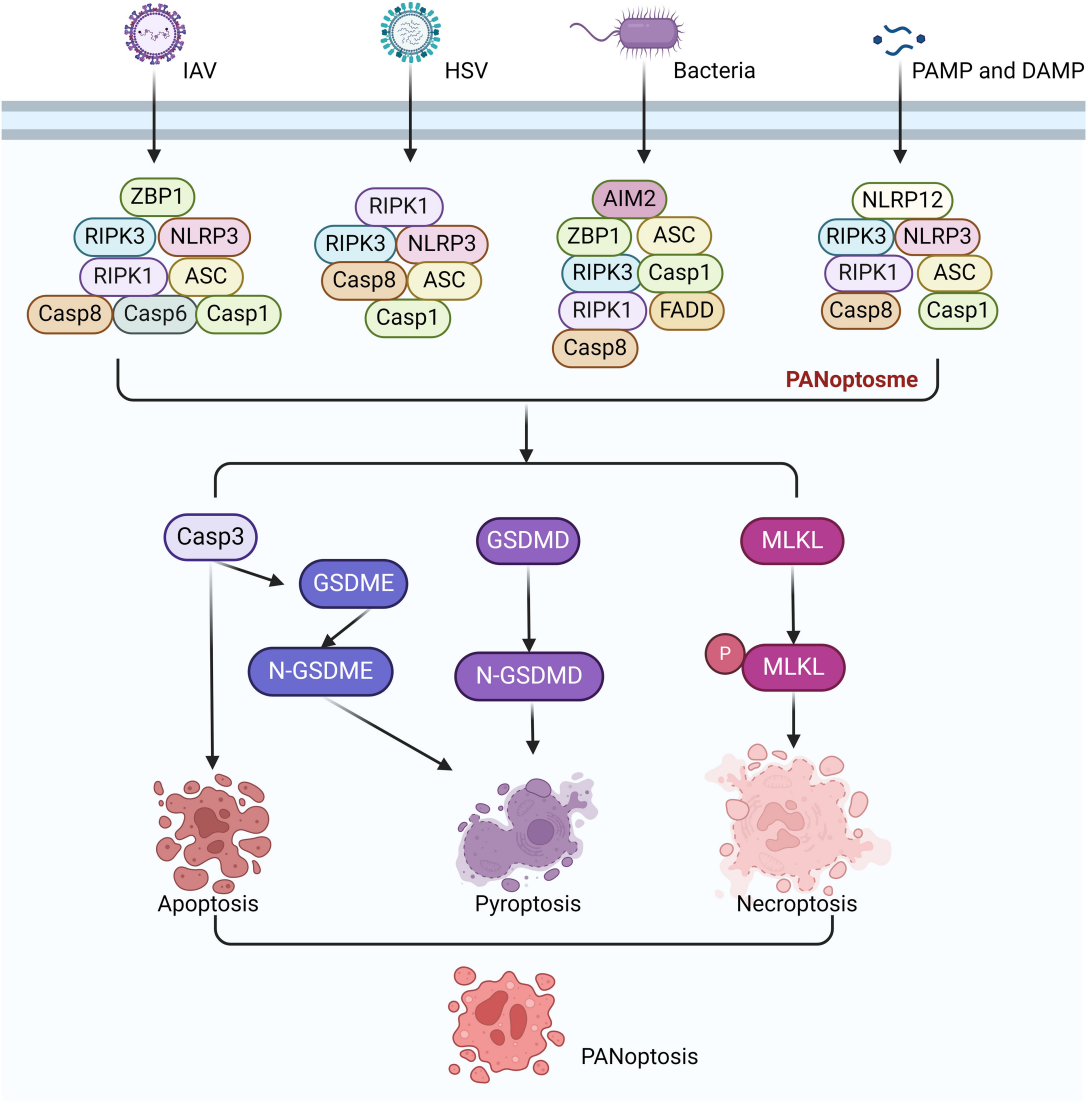
Signaling pathways of ZBP1-PANoptosome, RIPK1-PANoptosome, AIM2-PANoptosome and NLRP12-PANoptosome.

PANoptosis is an inflammatory PCD regulated by the PANoptosome. The PANoptosome is a complex composed of various proteins that play a central role in the process of PANoptosis (6). The classic PANoptosome is mainly composed of three parts: sensor proteins, adapter proteins and catalytic effectors. The sensor proteins include Z-DNA binding protein 1 (ZBP1), NOD-like receptor family containing pyrin domain 3, (NLRP3), and absent in melanoma 2 (AIM2), which can sense pathogen-associated molecular patterns (PAMPs) and damage-associated molecular patterns (DAMPs). The adapter proteins include apoptosis speck containing CARD (ASC) and Fas-associated protein with death domain (FADD), which help to transmit the signals from the sensor proteins to downstream effector molecules. The catalytic effector molecules include receptor-interacting serine/threonine-protein kinase 1 (RIPK1), receptor-interacting serine/threonine-protein kinase 3 (RIPK3), CASP1, and CASP8, which are involved in the execution of PANoptosis. Several types of PANoptosome with different sensors and regulatory factors have been identified, including ZBP1-PANoptosome, RIPK1-PANoptosome, AIM2-PANoptosome and NLRP12-PANoptosome (15) (**Figure 3**).

### 3.1 ZBP1-PANoptosome

The ZBP1 PANoptosome was the first PANoptosome to be discovered and identified. The discovery of the ZBP1-PANoptosome is related to Influenza A virus (IAV) infection. ZBP1 is an innate immune receptor that can recognize viral Z-DNA/RNA and induces cell death and inflammatory responses in infected cells (16). It ultimately induces PANoptosis in infected cells (13). The ZBP1-PANoptosome mainly consists of ZBP1, RIPK3, NLRP3, ASC, CASP8, CASP6, RIPK1, and FADD. The activation of the ZBP1-PANoptosome is a highly ordered and complex cascade reaction (17, 18). Further study found that its activation mainly includes: the recognition and activation of ZBP1, the assembly of the PANoptosome and the initiation of PANoptosis. During IAV infection, ZBP1 serves as a specific sensor that can recognize viral RNA, providing the initial signal for its activation. Subsequently, ZBP1 recruits RIPK3 and CASP8 through its RHIM domain to form the ZBP1-PANoptosome. This complex includes RIPK3, RIPK1, CASP8, FADD, ASC, NLRP3, and CASP1, which together form a molecular scaffold that allows the key molecules of pyroptosis, apoptosis, and necroptosis pathways to couple and bind. The PANoptosome activates the NLRP3, leading to the activation of CASP1 and the cleavage of GSDMD or GSDME, which then form pores in the plasma membrane, driving pyroptosis (18). In some cells with high expression of GSDME, pyroptosis can also be induced by CASP3-mediated GSDME cleavage after PANoptosome activation. However, it should be noted that in cells that do not express GSDME or where only CASP1 is activated, pyroptosis is induced through GSDMD cleavage (19). CASP8 is activated in the PANoptosome complex, which in turn activates downstream CASP3 and induces apoptosis. RIPK1 and RIPK3 interact in the PANoptosome complexs, leading to the phosphorylation of MLKL and the formation of MLKL pores, which drive necroptosis. Later research found that Caspase-6 (CASP6) was identified as a component of the complex that promotes the interaction between ZBP1 and RIPK3. Mice lacking CASP6 are more susceptible to infection with IAV (20).

### 3.2 RIPK1-PANoptosome

The activation process of the RIPK1-PANoptosome involves multiple steps and molecular interactions. Among them, the most critical molecule is RIPK1, which is a key signal transduction molecule that plays a central role in cell death and inflammation (21). Transforming Growth Factor-beta Activated Kinase 1 (TAK1) exists upstream of RIPK1 (5). It plays a key role in regulating NLRP3 inflammasome stillness and cell homeostasis (22). In TAK1-deficient macrophages, innate immune primes drive RIPK1-mediated PANoptosis (23). Upon infection of macrophages with Yersinia, TAK1 activity is suppressed, leading to the activation of RIPK1. Subsequently, RIPK1 forms the PANoptosome complex by recruiting RIPK1, RIPK3, NLRP3, ASC, CASP1, and CASP8. The formation of PANoptosome promotes downstream signaling and leads to PANoptosis (23, 24). The downstream molecular characteristics of PANoptosome are the activation of CASP1, CASP8, CASP3, the phosphorylation of MLKL and the cleavage of GSDMD or GSDME. However, compared with ZBP1-PANoptosome-mediated PANoptosis, the absence of RIPK1 does not completely prevent cell death. However, necroptosis significantly increases in the absence of RIPK1, suggesting that other molecules may be involved in the PANoptosis mediated by RIPK1-PANoptosome (23).

### 3.3 AIM2-PANoptosome

AIM2 is an intracellular receptor that recognizes DNA in cells, especially pathogen DNA (25). Upon recognition, AIM2 binds to the DNA through its HIN200 domain, thereby triggering the assembly and activation of the inflammasome (26, 27). In the case of infections with *Herpes Simplex* Virus-1 (HSV-1) and *Francisella novicida*, the formation of the AIM2-DNA complex promotes the binding of AIM2 to other proteins, assembling into a multiprotein complex known as the AIM2-PANoptosome. The AIM2-PANoptosome consists of AIM2, ZBP1, Pyrin, ASC, CASP1, CASP8, RIPK3, RIPK1, and FADD. The activation of the PANoptosome leads to PANoptosis in cells, thereby eliminating the infected cells (28). Similar to the ZBP1-PANoptosome, deletion of AIM2 can completely prevent PANoptosis (28). Interestingly, ZBP1 and pyrin are also involved in the formation of the AIM2-PANoptosome (28).

### 3.4 NLRP12-PANoptosome

NLRP12 is a member of the NLR family and is a NOD-like receptor. NLRP12 was first discovered in 2005 and can recognize intracellular pathogens or damage signals (29). Previous studies have found that the NLRP12 inflammasome can recognize *Yersinia pestis* (30). Heme is a central DAMP that can serve as a danger signal for disease. When red blood cells are damaged or lysed due to infection or other diseases, heme is released into the blood and can activate the innate immune system (31). When heme is combined with other PAMPs or cytokines, it drives NLRP12 to interact with other molecules such as ASC, CASP8, and RIPK3 to form the NLRP12-PANoptosome, which then drives PANoptosis (32). This combination mimics physiologically conditions, such as infection and cell damage occurring at the same time. The execution of pyroptosis involves the activation of CASP1, CASP8, CASP3 and RIPK3. These molecules induce cell death by cleaving substrates such as GSDMD, GSDME, and phosphorylation of MLKL. Recent research has found that NLRC5 also has similar functions to NLRC12. NLRC5 can recognize specific ligands such as heme and heme/cytokine combinations to drive inflammatory cell death, PANoptosis (33, 34).

Although four different PANoptosome complexes with distinct sensors have been identified, the precise triggers that activate these sensors and how they distinguish between different pathogens (such as viral and bacterial infections) remain unclear. Additionally, the assembly processes and mechanisms of action of different PANoptosome complexes under various stimuli are not fully understood. Although PANoptosis integrates the features of apoptosis, pyroptosis, and necroptosis, the interactions and transitional mechanisms between PANoptosis and these traditional cell death pathways remain unclear.

## 4 The role of pyroptosis in cancer treatment

It has been discovered that PANoptosis plays a role in a variety of diseases, including infectious diseases, cancer, neurological disorders, respiratory diseases and autoimmune diseases (7). Recently, the role of PANoptosis in cancer treatment is attracting more and more attention. In cancer treatment, PANoptosis can play a role in many aspects such as inducing tumor cell death, triggering a strong anti-tumor immune response, providing new therapeutic targets, overcoming drug resistance in tumor cells, regulating the tumor microenvironment, prognostic models and drug sensitivity.

### 4.1 PANoptosis is closely associated with various types of cancer

More and more evidences indicate that PANoptosis is closely related to tumorigenesis, carcinogenesis, and prediction of various cancers. It has been found that PANoptosis plays a role in various types of cancer, such as colorectal cancer, hepatocellular carcinoma (HCC), gastric cancer (GC), breast cancer (BC), clear cell renal cell carcinoma (ccRCC), melanoma, and glioma (**Table 1**).

**Table 1:** PANoptosis is induced various types of cancer.

Cancer types	Inducers	PANoptosis Activity	Mechanism	Reference
Colorectal cancer	AOM/DSS	High activity	IRF1 as an upstream regulator of PANoptosis	(35)
Lung cancer	PM2.5	High activity	Through the EIF4B/WTAP/m6A axis	(37)
Chronic large B-cell lymphoma	STING agonists	High activity	Through CASP 8/RIPK3/ASC PANoptosome	(38)
Adrenocortical carcinoma	CDK1 inhibitor CurE	High activity	CDK1 bind to the PANoptosome	(39)
Breast cancer	Atramacronoid A	High activity	through the CASP3/PARP-GSDMD-MLKL pathway	(40)
Gastric Cancer	Jolkinolide B; YBX1;	High activity	by activating CASP8; decreased YBX1 protein expression levels and impacting PANoptosis	(70, 103);
Esophageal cancer	Sulconazole	High activity	by Triggering Oxidative Stress and Inhibiting Glycolysis	(72)
Melanoma	IFN-γ and the NEI KPT-330	Moderate activity	ZBP1-mediated PANoptosis	(81)
**Acute Myeloid Leukemia (AML)**	TNF-α or IFN-γ	Low activity (resistance)	Integrated stress response activation suppresses PANoptosis.	(104)
**Hepatocellular Carcinoma (HCC)**	Sorafenib	Medium-low activity	Metabolic reprogramming modulates sensitivity	(105)

Studies have shown that PANoptosis is closely associated with the tumorigenesis of colorectal cancer and colon adenocarcinoma (COAD). IRF1 plays a key role in regulating various biological functions, including cellular responses related to tumorigenesis. Study has found that mice deficient in IRF1 are prone to developing colorectal tumors. In IRF1-deficient mice, the reduction of colon cell death is due to the deficiency of pyroptosis, apoptosis, and necroptosis. The study identified IRF1 as an upstream regulator of PANoptosis that induces PANoptosis during the development of colorectal cancer. Furthermore, it was found that low expression of IRF1 is associated with poor prognosis in patients with advanced colorectal cancer (35, 36).

The carcinogenesis of lung cancer is closely related to PM2.5 pollution. Recently, piR-27222 was identified as an oncogene that inhibits cell death in an m6A-dependent manner. Exposure to PM2.5 upregulates the expression of piR-27222 in lung cancer cells, which subsequently leads to reduced stability of Casp8 transcripts through the EIF4B/WTAP/m6A axis. Ultimately, this inhibits PANoptosis and promotes the carcinogenesis of lung cancer (37).

In Diffuse Large B-cell Lymphoma (DLBCL), the sterile α motif and HD domain containing protein 1 (SAMHD1) deletion activate stimulators of interferon genes (STING) expression. Overexpression of STING promotes the formation of the CASP 8/RIPK3/ASC PANoptosome, which further leads to MLKL phosphorylation, CASP3 cleavage and GSDME cleavage. Ultimately, this induces PANoptosis in cells and inhibits tumor growth in DLBCL (38).

In adrenocortical carcinoma (ACC), cyclin-dependent kinase-1 (CDK1) is highly expressed. The CDK1 inhibitor CurE can inhibit tumors by regulating epithelial-mesenchymal transition (EMT), G2/M phase transition, and PANoptosis. Mechanistically, CDK1 regulates PANoptosis in ACC cells through binding to the PANoptosome in a ZBP1-dependent manner (39).

In breast cancer, the natural sesquiterpene lactone compound Atramacronoid A (AM-A) can induce PANoptosis in breast cancer through the CASP3/PARP-GSDMD-MLKL pathway (40).

These results indicate that PANoptosis is involved in the tumorigenesis and carcinogenesis of various cancers. PANoptosis can suppress tumors by inducing malignant cell death in some cases. However, certain cancers can evade it (41). The molecular switches that determine these opposite outcomes remain unknown. Moreover, inducing PANoptosis in cancer cells may trigger unintended inflammation or drug resistance.

However, the expression of PANoptosis-related genes in these cancers is relatively complex (**Table 2**). There is still much unknown about its regulatory mechanisms in cancer. Further studies are needed to discover the specific associations between PANoptosis and cancer.

**Table 2:** PANoptosis-related gene expression levels across cancers.

Cancer types	Key PANoptosis-Related Genes Expression Level	Activity Context	References
Kidney Renal Clear Cell Carcinoma (KIRC)	CASP4(↑), LY964(↑), TLR34(↑), ZBP14(↑), IRF14(↑)	High PANoptosis activity correlates with better immunotherapy response and prognosis	(47)
Biliary Tract Cancer (BTC)	CASP1(↑), GSDMD(↑), IRF1(↑), PARP1(↑), RIPK1(↑)	Moderate-high activity; high PRG expression linked to poor prognosis	(106)
Glioma	IDH1(↑), TP53(↑), ATRX(↑), EGFR(↑), NLRP3(↑)	Heterogeneous activity; subtype-dependent (high activity in IDH-wildtype tumors)	(106)
Hepatocellular Carcinoma (HCC)	CASP8 (↑), FADD (↑), GSDMD (↑), NLRP3 (↓), AIM2 (↓)	Complex regulation; metabolic pathways (TAK1/Ragulator) influence PANoptosis	(107)
Cutaneous Melanoma (CM)	ZBP1 (↓), MAP3K7 (↓), RBX1 (↓)	Inducible activity; low expression correlates with poor survival and immune evasion	(108)
Acute Myeloid Leukemia (AML)	RIPK1(↓), MLKL(↓)	Resistance due to ISR activation; requires combinatorial targeting	(47, 108)
Colorectal Cancer (CRC)	NFS1 (↓), ADAR1 (↓), ZBP1 (↑)	High activity predicts chemotherapy sensitivity and immune activation	(47, 106)

The critical role of PANoptosis in various cancers depends on the specific genetic, molecular, and microenvironmental characteristics of each cancer type, as well as the potential of PANoptosis to either suppress or promote tumor growth in these contexts. Certain cancers possess specific genetic mutations or altered signaling pathways that make them more susceptible to PANoptosis induction. For instance, in lung cancer, FADD, a key adaptor in PANoptosis, serves as a significant risk factor. The expression levels of certain PANoptosis-related genes (TRADD, NLRC4, RIPK1, MLKL, and PSTPIP) are associated with the prognosis of lung adenocarcinoma (42). The expression levels of specific molecular targets involved in PANoptosis pathways may vary across different cancer types (**Table 2**). For example, in tumor samples, the expression level of GSDMD shows a positive correlation with CD8+ T cell levels, suggesting that cancers with higher GSDMD expression may respond better to PANoptosis induction (43).

### 4.2 The role of PANoptosis in the tumor microenvironment

**Figure 4:**
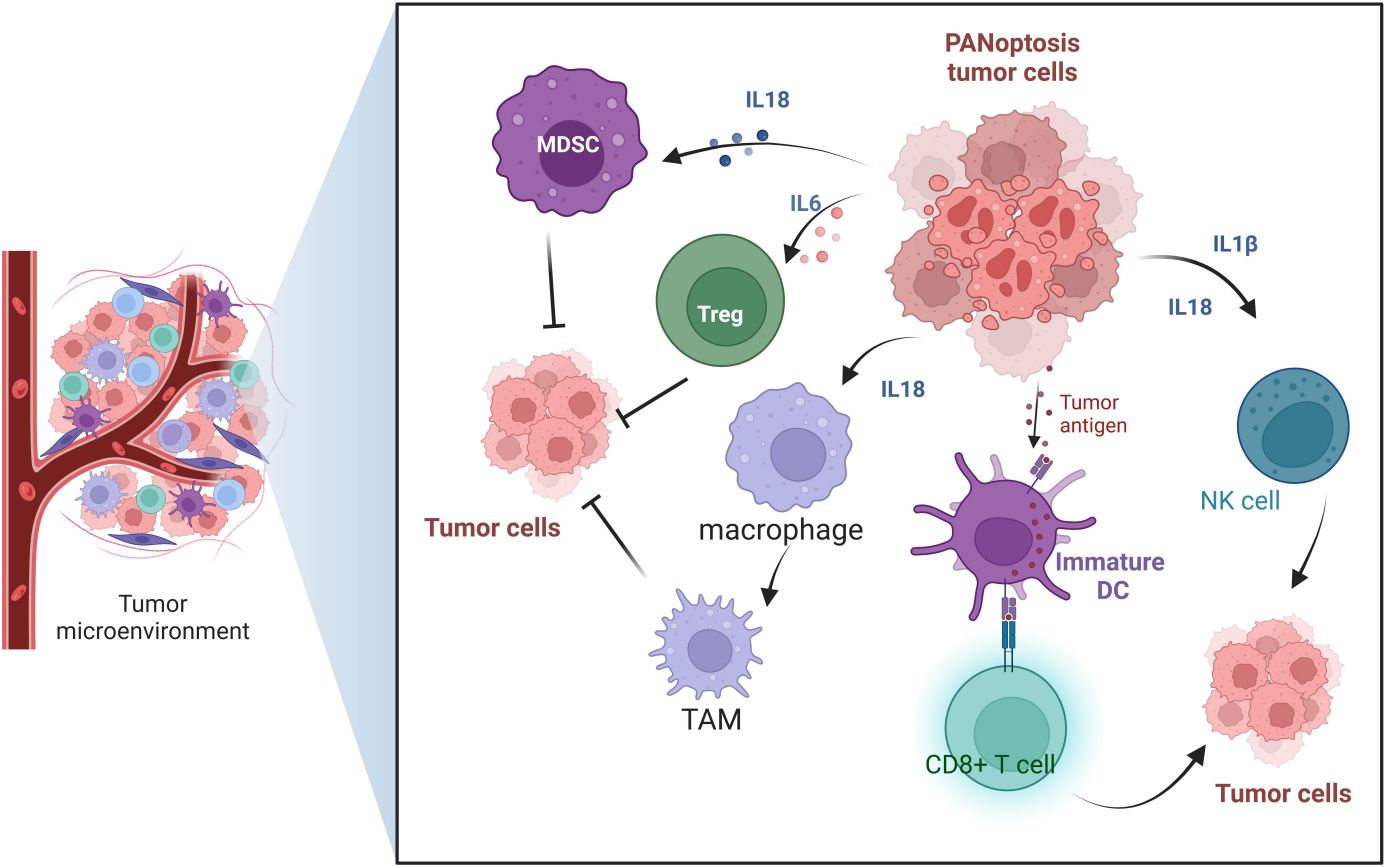
The role of PANoptosis in the tumor microenvironment.

The tumor microenvironment (TME) plays a crucial role in the tumorigenesis, invasion, metastasis, and response to therapy (44). TME is a complex ecosystem composed of tumor cells, immune cells, stromal cells, extracellular matrix and various cytokines and signaling molecules (45). It was found that PANoptosis affects the interaction of these components, which in turn affects tumorigenesis and metastasis. In the TME, the role of PANoptosis is gradually becoming a hot topic of research. PANoptosis not only acts directly on tumor cells, but also exerts anti-tumor effects by affecting TME. On the one hand, cellular contents released during PANoptosis, such as various DAMPs, can activate multiple immune cells, such as dendritic cells(DCs) and macrophages, thereby enhancing the anti-tumor immune response. On the other hand, PANoptosis can also regulate cytokines and chemokines in the TME, further affecting the recruitment and activation of immune cells (**Figure 4**).

#### 4.2.1 PANoptosis regulates a variety of immune cells in TME

In TME, PANoptosis can regulate a variety of immune cells, including macrophages, DCs, regulatory T cells, and natural killer (NK) cells. PANoptosis plays an important role in regulating the function of immune cells in the tumor TME through multiple mechanisms. PANoptosis can affect tumor-associated macrophages (TAMs) (46–48). PANoptosis may affect the polarization of TAMs and regulate their role in anti-tumor immunity by changing the signaling molecules in TME (49). M1 macrophages have anti-tumor effects, while M2 macrophages may promote tumor growth and metastasis. PANoptosis can also regulate dendritic cells (50). After inducing PANoptosis of tumor cells in TME, tumor antigens are released and DCs are activated, thus enhancing the activity and proliferation of T cells. This process helps initiate and maintain an anti-tumor immune response. PANoptosis can regulate the subtypes of immune cells. In addition, PANoptosis can enhance the function of NK cells (51). Cytokines released during PANoptosis, such as IL-1β and IL-18, can activate NK cells and enhance their ability to kill tumor cells, thus improving the effectiveness of anti-tumor immune response (52). By inducing the death of tumor cells, tumor cells can be recognized and activated by NK cells to promote their killing effect. However, tumors can inhibit NK cell activity by secreting inhibitory factors (such as TGF-β) or by expressing apoptosis-related molecules. PANoptosis also regulates the function of T cells (53). PANoptosis can activate CD8+ T cells and enhance their recognition and killing of tumor cells (47). In addition, it may affect the function of regulatory T cells (Tregs), reduce their immunosuppressive effect, and thus improve anti-tumor immunity (54) (**Figure 4**). In summary, PANoptosis plays a multi-faceted role in the TME. It enhances anti-tumor immune by activating and regulating a variety of immune cells, and provides a new strategy and target for cancer treatment.

PANoptosis not only directly kills tumor cells but also enhances anti-tumor immunity by releasing DAMPs. These DAMPs promote the maturation of dendritic cells, the polarization of macrophages, and the infiltration of immune cells such as CD8+ T cells and NK cells into the TME. This forms a positive feedback loop, further activating the immune system to fight against the tumor and potentially establish lasting immune memory and protection.

#### 4.2.2 Cytokines released by PANoptosis affect the TME

The activation of PANoptosis can release a variety of cytokines, which play an important role in the tumors. PANoptosis has the characteristics of three forms of programmed cell death: pyroptosis, apoptosis, and necroptosis. Among them, pyroptosis and necrosis are inflammatory forms of cell death. When cells undergo pyroptosis, they release various cytokines such as IL-1β, IL-18, and HMGB1 (55). Necroptotic cells can release regulatory cytokines, which can directly stimulate the proliferation of neighboring cells and potentially promote tumor growth. The release of these cytokines and DAMPs helps to shape the tumor microenvironment, affecting the immune surveillance and immune evasion of tumor cells.

IL-1β and IL-18 both belong to the IL-1 cytokine family. They are important cytokines in the tumor microenvironment and play a key role in regulating immune responses and tumorigenesis (56). IL-1β is a pro-inflammatory cytokine, mainly produced by immune cells such as macrophages and DCs. The role of IL-1β in the TME is mainly reflected in two aspects. On the one hand, it promotes the tumorigenesis by inducing local inflammatory responses in the TME. On the other hand, it plays an immunomodulatory role. IL-1β has a regulatory effect on various immune cells such as T cells, NK cells, and macrophages (57). IL-1β can promote the survival and proliferation of T cells, and it can also promote the maturation of DCs, thereby enhancing T cell-mediated adaptive immune responses (58).

IL-18 plays a multifaceted role in the TME. It not only directly activates immune cells but also enhances anti-tumor immune responses by altering the state of the TME (52). It activates immune cells mainly by activating natural killer (NK) cells and cytotoxic T cells (CTLs) to enhance their killing ability to tumor cells (59). IL-18 can also promote the proliferation and activation of tumor-specific T cells and enhance their immune surveillance against tumors (59). IL-18 works in synergy with other cytokines to promote Th1-mediated immune responses. Additionally, IL-18 can also regulate the number of regulatory T cells (Tregs) and tumor-associated macrophages (TAMs) (60).

IL-1α, like IL-1β, belongs to the IL-1 family. IL-1α can regulate the composition and function of immune cells in the TME. For example, IL-1α can promote the accumulation of regulatory T cells (Tregs) and myeloid-derived suppressor cells (MDSCs). IL-1α can also induce the polarization of TAMs towards the M2 phenotype, while suppressing the activity of effector T cells and NK cells, further promoting tumor immune evasion and growth (61, 62). IL-1α not only affects immune cells but can also directly promote the proliferation and survival of certain tumor cells by upregulating the expression of genes associated with tumorigenesis and metastasis (63) (**Figure 4**).

#### 4.2.3 PANoptosis interacts with immune checkpoint pathways

PANoptosis, which integrates multiple cell death pathways, dynamically interacts with PD-1/PD-L1 immune checkpoint signaling through mechanisms that shape antitumor immunity and therapeutic responses. PANoptosis triggers the release of DAMPs and cytokines, which modulate PD-L1 expression in both tumor cells and immune cells (64). Additionally, PANoptosis remodels the TME to either enhance or counteract PD-1/PD-L1-mediated immunosuppression. For example, in melanoma, GSDME-mediated PANoptosis promotes antigen presentation and dendritic cell maturation, counteracting PD-L1-driven T cell exhaustion (65). PANoptosis also exhibits synergistic effects with immune checkpoint inhibitors (ICIs). In glioblastoma (GBM), cinobufagin combined with anti-PD-1 therapy demonstrates significant synergistic effects and prolongs survival in GBM models (66). PANoptosis and the PD-1/PD-L1 pathway display a bidirectional regulatory relationship. While PANoptosis can improve ICIs efficacy by reshaping the TME, it may also drive resistance through PD-L1 upregulation. Targeting PANoptosis components in combination with ICIs represents a promising strategy, particularly in cancers with high PD-L1 expression or immunosuppressive TMEs.

### 4.3 The role of PANoptosis in drug resistance of tumors

Drug resistance is a complex and critical issue in cancer treatment, involving various mechanisms. These mechanisms usually do not act independently, but are interrelated and work together to promote the resistance of tumor cells to chemotherapy drugs. One important mechanism is that cancer cells alter the signaling pathways of programmed cell death, enabling them to resist drug-induced cell death (67). Programmed cell death such as apoptosis, necroptosis and pyroptosis have been reported to be closely related to drug resistance in tumors. The recently discovered PANoptosis also plays a significant role in tumor drug resistance.

Pyroptosis involves the complex interaction of multiple signaling pathways and molecular mechanisms, including the PANoptosome complex, Caspases family proteins (such as CASP1, CASP8, CASP3, etc.) and gasdermin family members (such as GSDMD, GSDME). The expression levels and activity changes of these molecules in drug-resistant tumor cells may directly affect the sensitivity of tumor cells to chemotherapeutic drugs.

Cysteine desulphurase NFS1 is a rate-limiting enzyme in iron-sulfur (Fe-S) cluster biogenesis. In colorectal cancer, NFS1 is strongly associated with poor survival and low sensitivity to chemotherapy in CRC patients. NFS1 deletion enhances the sensitivity of CRC cells to oxaliplatin. Mechanistically, oxaliplatin stimulates NFS1 phosphorylation and prevents CRC cells from PANoptosis, resulting in drug resistance. In contrast, the absence of NFS1, in synergy with oxaliplatin, can induce PANoptosis, thereby improving the therapeutic efficacy of oxaliplatin chemotherapy (68). In another study, it was found that high expression of the m6A methyltransferase Wilms tumor 1-associating protein (WTAP) in CRC patients is associated with poor prognosis and reduced benefit from standard chemotherapy. In CRC cells, inhibition of WTAP expression suppressed PANoptosis and thus inhibited oxaliplatin efficacy (69). In GC cells, YBX1 can inhibit the PANoptosis, which leads to oxaliplatin resistance in GC cells. Mechanistically, PPM1B and USP10 interact with YBX1, resulting in the dephosphorylation of YBX1. This affects the USP10-mediated deubiquitination of YBX1, ultimately inducing PANoptosis in GC cells and resistance to oxaliplatin (70). In laryngeal cancer-resistant cells, the level of lncRNA FLJ20021 is elevated. LncRNA FLJ20021 inhibits PANoptosis by regulating CDK1 through the ZBP1 pathway. Silencing lncRNA FLJ20021 can significantly increase the sensitivity of laryngeal cancer-resistant cells to cisplatin (71).

The PANoptosome is involved not only in resistance to chemotherapy drugs but also plays a significant role in radiation resistance. Sulforaphane has broad anticancer effects and can inhibit the growth of esophageal cancer cells. Sulforaphane triggers oxidative stress and suppresses glycolysis to induce PANoptosis in esophageal cancer cells, thereby enhancing their sensitivity to radiation (72).

Tumor drug resistance is a complex process involving multiple factors and mechanisms (73). A thorough understanding of the mechanisms of PANoptosis in drug resistance is crucial for developing new treatment strategies and improving the effectiveness of cancer treatment.

### 4.4 Prediction of cancer prognosis by PANoptosis

PANoptosis, as a novel form of programmed cell death, plays an important role in predicting cancer prognosis. Studies have shown that the expression of PANoptosis-related genes is closely associated with the prognosis of various types of cancer. The prognostic prediction of PANoptosis genes in cancer holds significant clinical importance, as it can provide vital information for the early diagnosis of cancer, selection of treatment options, and assessment of prognosis.

A variety of PANoptosis-related genes (PRGs) are associated with the tumorigenesis and prediction of colorectal cancer. Through the analysis of multiple databases, researchers have identified five key PRGs related to the carcinogenesis of CRC, including BCL10, CDKN2A, DAPK1, PYGM, and TIMP1 (74).

In breast cancer, the natural sesquiterpene lactone compound Atramacronoid A (AM-A) can induce PANoptosis in breast cancer through the CASP3/PARP-GSDMD-MLKL pathway (40). Additionally, PANoptosis-related genes were obtained from the Gene Expression Omnibus (GEO) and The Cancer Genome Atlas (TCGA) databases, and prognostic models were constructed using COX regression and LASSO regression to identify PANoptosis-related genes with prognostic value. The results showed that the molecular aggregation and prognostic characteristics based on PANoptosis have the potential to predict the survival of breast cancer patients (75). A similar systematic analysis of 37 Panoptosis-related genes found that XIAP is a functional oncogene in breast cancer (76). Another research group also identified 10 PANoptosis-related genes, and further analysis found that CHMP2B is highly expressed in tumor tissue and that CHMP2B adversely affects the survival of BC patients (77).

In gastric cancer, the authors analyzed the molecular, clinical, and immunological characteristics of the PANoptosis pattern in 1,316 GC patients. They constructed a scoring system called PANscore to quantify the PANoptosis pattern in individual GC patients. The results showed that the characterization of PANoptosis can predict survival rates and immune therapy responses in gastric cancer (78). In another study, the expression patterns and immunological characteristics of PANoptosis-related genes in gastric cancer were also investigated, and the PANoptosis pattern could potentially serve as a stratification tool for patient risk assessment (79). Recently, the PANoptosis-related risk score (PANS) has been developed, which is constructed from genes associated with PANoptosis and classifies patients into different PAN subtypes. Subsequently, the correlation between PANS and GC prognosis, TME, efficacy of immunotherapy and sensitivity to chemotherapeutic drugs was analyzed, the results showed that the PANoptosis subtypes can predict the prognosis and immune effects of gastric cancer (80).

By constructing a risk model based on PANoptosis genes, it is possible to accurately assess the prognosis and immune status of patients, providing important information for clinical decision-making. At the same time, these genes may also become potential therapeutic targets, opening up new avenues for cancer treatment. In the future, with the advancement of research and technology, the role of PANoptosis genes in cancer prognosis prediction and treatment will be more fully explored and utilized.

## 5 Targeting PANoptosis as cancer treatment strategies

**Figure 5:**
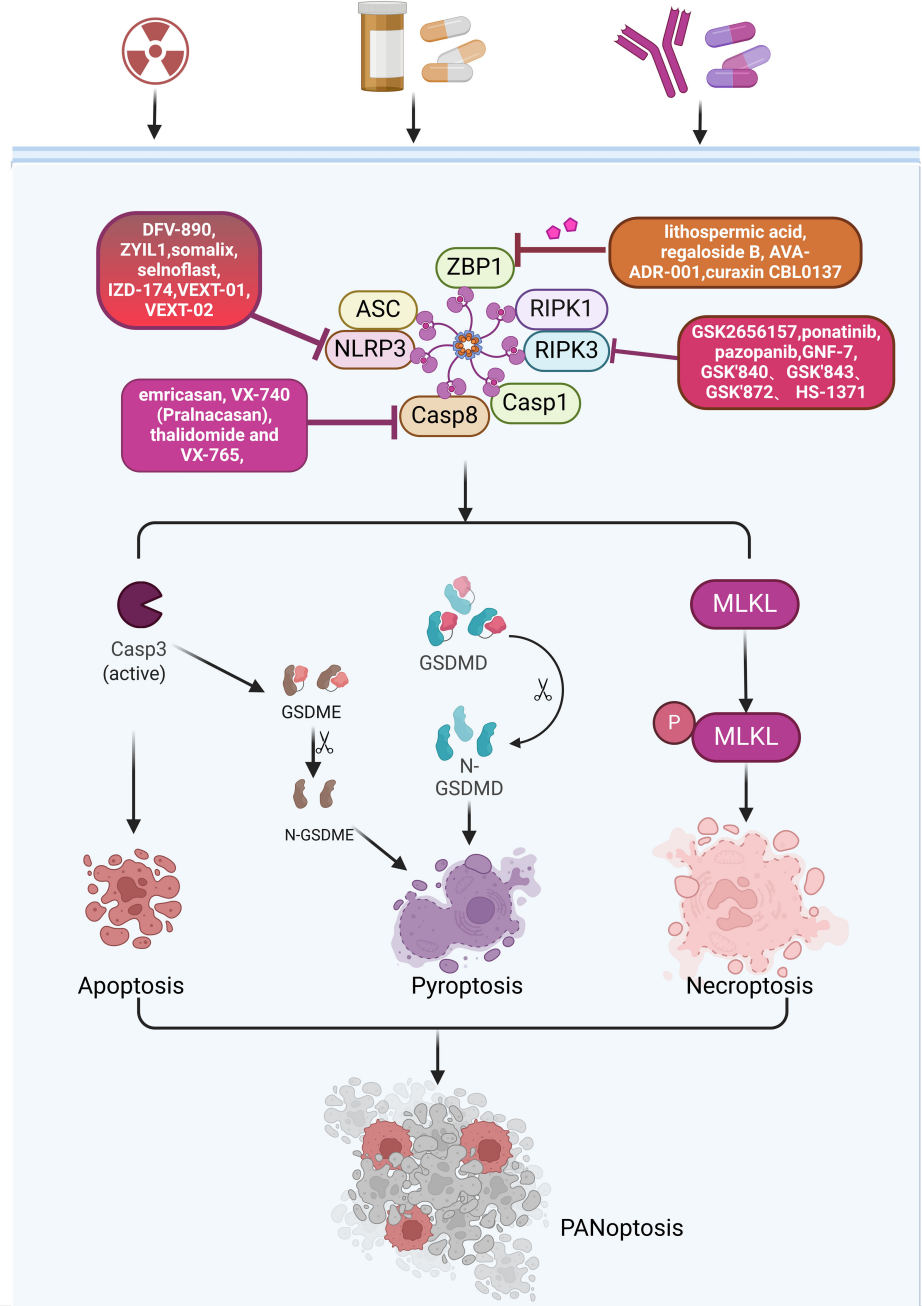
Various inhibitors targeting key proteins in the PANoptosis pathway.

In cancer treatment, targeting key molecules in PANoptosis may become an emerging therapeutic approach. For example, by targeting the upstream signal transduction pathways, receptors, or by targeting PANoptosome, it is possible to effectively block the initiation of the three types of cell death pathways associated with PANoptosis. This provides new options for cancer treatment, especially when tumor cells develop resistance to traditional chemotherapy drugs. Activating multiple cell death pathways may help overcome this limitation. A variety of small molecule compounds and nanoinducers have been developed. These compounds can induce tumor cells to undergo PANoptosis and suppress tumor growth through various mechanisms. Some of these compounds have already entered the clinical trial stage and have shown significant effects (**Figure 5**).

### 5.1 Targeting ZBP1

ZBP1, as a sensor protein in the innate immune, triggers and regulates cell death through its specific molecular mechanisms during PANoptosis. In colorectal cancer and melanoma, ADAR1 promotes tumorigenesis by inhibiting ZBP1-mediated PANoptosis (81). Some tumors rely on ADAR1 to evade immune surveillance, hence targeting ADAR1 can be used for anti-cancer therapy. It has been discovered that lithospermic acid and regeloside B are potential inhibitors that interact with the Zα domain of ADAR1. AVA-ADR-001 is a potential selective inhibitor of the ADAR1p150 subtype. ZBP1 activator curaxin CBL0137 (82), which effectively activates ZBP1 by triggering Z-DNA formation in cells, activates ZBP1 and ZBP1-mediated necrotic apoptosis (82) (**Figure 5**).

### 5.2 Targeting NLRP3

NLRP3-mediated PANoptosis plays a variety of roles in driving carcinogenesis, immunosuppression, and metastasis (83). Small molecule inhibitors targeting NLRP3 have been widely reported, many of which have been applied in clinical and preclinical settings (84). Several companies already have NLRP3 inhibitors in the clinic (85). These inhibitors include phage11, phage1, and IND-enabling stages. The phage11 inhibitors include DFV-890, ZYIL1, OLT-1177 and VTX-2735. The phage1 inhibitors include somalix, selnoflast, IZD-174, NT-0249, NT-0796 and VTX-3232. The IND-enabling stage includes various inhibitors such as VEXT-01 and VEXT-02. Although an increasing number of inhibitors are being developed, most of them are still in the early stages of clinical research (**Figure 5**). Whether it will eventually be clinically useful has not been reported, and the role of these inhibitors in cancer treatment needs further study (86). In addition to these drugs already in clinical studies, there are a number of NLRP3 inhibitors in preclinical and laboratory development stages. Based on their structural characteristics, they can be divided into the following six categories: MCC950 analogues, glyburide derivatives, triazine ketone derivatives, dorsoapine derivatives, thiazolone compounds, and α, β-unsaturated carbonyl compounds (86).

### 5.3 Targeting RIPK1 and RIPK3

RIP is a serine/threonine protein kinase, with RIPK1 and RIPK3 being the most extensively studied (87). RIPK1 is one of the important molecules constituting the RIPK1-PANoptosome, which also includes ASC, CASP1, CASP8 and death-associated structural domain protein (FADD). RIPK1 participates in the initiation and execution of PANoptosis by interacting with these molecules. RIPK3 is a core molecule in the process of necroptosis, which induces cell membrane rupture and cell death through phosphorylation and activation of MLKL (88). In clinical applications, compounds targeting RIPK1 and RIPK3 may induce or inhibit PANoptosis by affecting the function of these proteins. Among them, inhibitors targeting RIPK1 include Necrostatin, Necrostatin-1s, ZJU-37, GSK2606414 and GSK2656157, ponatinib, pazopanib, GNF-7 and KW-2449, tozasertib and its derivative Cpd-71, GSK’481 and GSK2982772 (**Figure 5**). Among these inhibitors, PK68 can directly block the kinase activity of RIPK1 (89). Pre-treatment with PK68 significantly inhibits the metastasis of mouse melanoma cells and lung cancer cells (90). GSK′547 is a derivative of GSK’963 and has higher metabolic stability (87). RIP1 is highly expressed in TAMs in pancreatic ductal adenocarcinoma (PDA). Treatment of TAMs in pancreatic ductal adenocarcinoma with GSK′547 leads to the activation of cytotoxic T cells and the differentiation of T helper cells into a mixed Th1/Th17 phenotype, ultimately inducing tumor immunity (91). A variety of inhibitors have also been developed for RIPK3, such as GSK’840, GSK’843, GSK’872, HS-1371, and AZD5423. Currently, preclinical studies are being conducted on inhibitors including GW440139B, ZINC71828321, ZINC72474191, and ZINC72454060 (92) (**Figure 5**). Although there has been some research on these inhibitors, reports on their clinical applications are still limited, and very few studies have reported their role in cancer treatment. Further in-depth research in these areas is needed in the future.

### 5.4 Targeting various caspases

Caspases play a multifaceted role in PANoptosis. They not only directly involve in the execution of cell death, but also affect the process of PANoptosis by regulating inflammatory response and interacting with other cell death pathways. By targeting specific Caspases, such as CASP1, CASP3, CASP6, and CASP8, the PANoptosis of tumor cells can be regulated (93).

CASP8 is not only involved in the regulation of a variety of cell death pathways, but may also affect the TME and immune response, which makes CASP8 a potential target for cancer therapy (94). There are currently several compounds that induce tumor cell death by targeting CASP8. Shikonin is a natural naphthoquinone compound that has been reported to induce tumor cell death through various mechanisms such as apoptosis, pyroptosis and necroptosis (95). In osteosarcoma, Shikonin promotes doxorubicin-induced apoptosis by upregulating CASP3 and CASP8 in osteosarcoma (96). In gastric cancer, Shikonin induces pyroptosis by inducing CASP3-mediated GSDME cleavage (95). Although Shikonin has shown antitumor potential in laboratory studies, its safety and efficacy in clinical applications still need to be verified through clinical trials. Small molecule CaspPro, which activates CASP8, can directly bind to CASP8 and enhance its activation. It can stimulate TRAIL-induced apoptosis in cancer cells (97). Four drugs targeting CASP1, emricasan, VX-740 (Pralnacasan), thalidomide and VX-765, have been tested in the clinic (10) (**Figure 5**).

Although a variety of Caspases inhibitors have been designed and synthesized, only a few have entered clinical trials. Due to poor efficacy, low specificity or side effects, no Caspases inhibitor has been successfully used in the clinic.

### 5.5 Other strategies to induce PANoptosis

**Figure 6:**
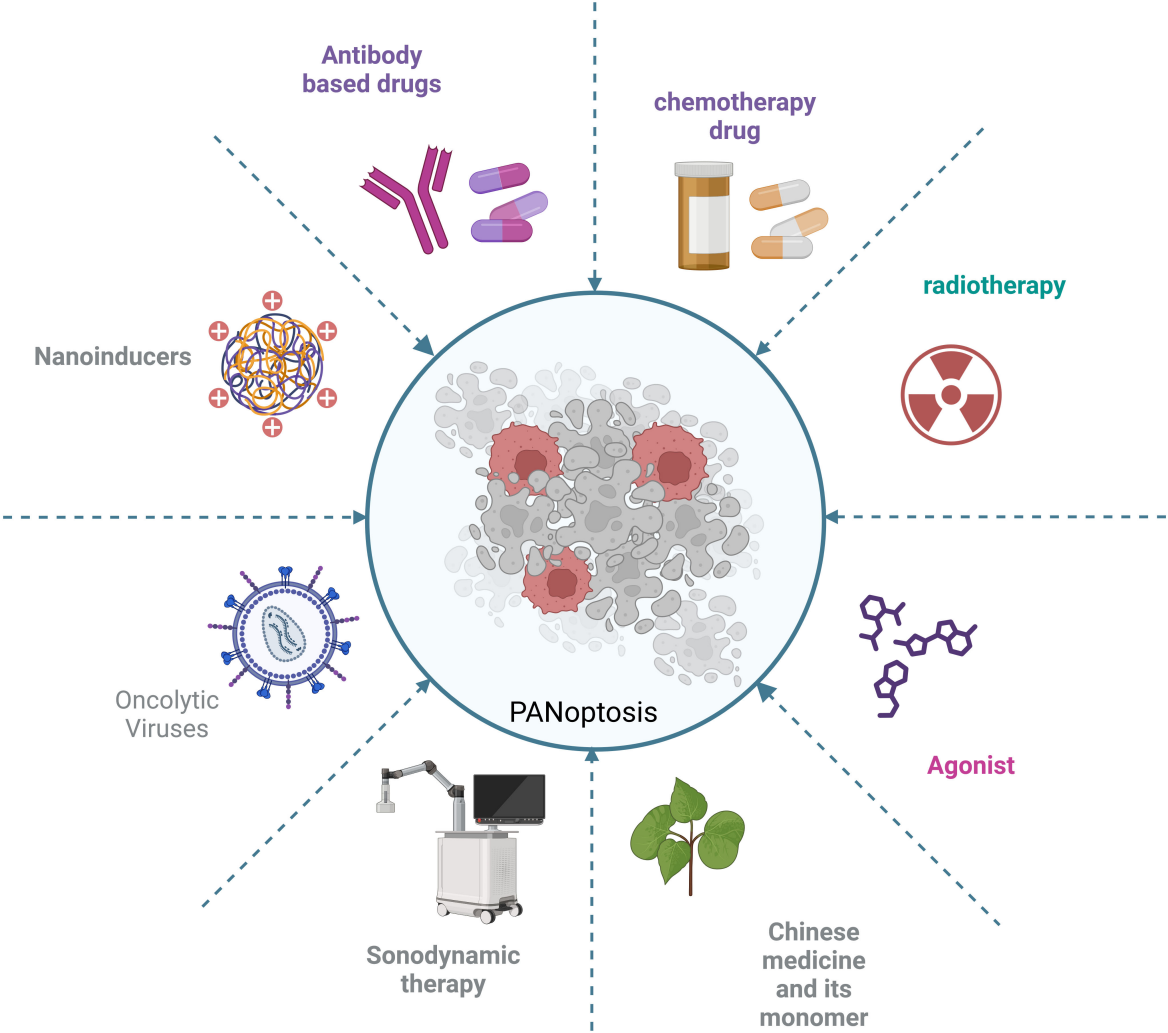
Various strategies targeting PANoptosis in tumor cells.

Although targeting key proteins in the PANoptosis pathway is a major strategy in current cancer treatment, there are currently many other strategies that inhibit tumor cell growth and have significant therapeutic effects through PANoptosis. These strategies include nanoinducers, viral vectors, targeting metabolic enzymes and targeting the tumor microenvironment for immunotherapy (**Figure 6**).

Nanotechnology is increasingly being used in cancer treatment. Nanoinducers have attracted much attention because of their ability to improve drug delivery efficiency, enhance drug targeting and reduce side effects. Nanoinducers have also shown potential applications in the study of PANoptosis (53, 98). In liver cancer, the efficiency of hepatocellular carcinoma treatment is very low, hence there is an urgent need for new approaches for liver cancer treatment, such as nanoinducers. The authors have developed an ultra-small Bi2Sn2O7 nanoenzyme with ultrasound-amplified multi-enzyme-mimicking properties. This nanoenzyme induces the production of reactive oxygen species (ROS) by disrupting the mitochondrial function of tumor cells, ultimately inducing PANoptosis in hepatocytes for the treatment of hepatocellular carcinoma and inhibition of lung metastasis (98). In another study, the authors developed a strategy triggered by ultrasound nanomedicine using nano/genetically engineered extracellular vesicles extracellular vesicles. The strategy induces immunogenic cell death by triggering PANoptosis in tumor cells, ultimately leading to the activation of sufficient antigen-specific T cells to kill the tumor (53). Sonodynamic therapy (SDT) is an innovative, non-invasive and precise approach to tumor treatment. Its therapeutic effect is closely related to the ability of sonosensitizers to generate reactive oxygen species (ROS). A novel nano-enzyme acoustic sensitizer @BiOI@Pt-PVP (BBP) has been developed. This sonosensitizer can enhance the efficiency of ROS generation under ultrasound activation, exacerbate cellular oxidative damage and ultimately trigger PANoptosis and ferroptosis in tumor cells (99). These approaches combine nanotechnology with tumor immunotherapy techniques, giving PANoptosis a broader application in cancer treatment.

Oncolytic Viruses (OVs), as a new cancer treatment method, have become an important research direction in tumor immunotherapy because of their ability to selectively infect and kill tumor cells while retaining normal cells (100). Another strategy is to inhibit tumor growth by inducing PANoptosis in tumor cells through oncolytic viruses. However, the immunogenicity of tumor treatments induced by OVs is relatively weak, leading to suboptimal therapeutic effects. Therefore, the authors developed OVs based on HSV-1 (oHSV) to trigger ZBP1-mediated PANoptosis in tumor cells, and the results showed effective antitumor immunogenicity (101).

### 5.6 Safety concerns and possible off-target effects of PANoptosis activation in cancer therapy

While activating PANoptosis holds therapeutic potential in cancer treatment, it also carries safety risks and potential off-target effects. The molecular components of PANoptosis are not exclusive to cancer cells. Drugs or strategies designed to activate PANoptosis in cancer therapy may induce toxicity in normal tissues, leading to cell death or dysfunction in healthy cells. Studies indicate that PANoptosis activation in immune cells or epithelial cells could cause immunosuppression or organ damage. For example, ZBP1-mediated PANoptosis in DNA-damaged fibroblasts has been shown to exacerbates chemotherapy-induced side effects (102). Additionally, PANoptosis activation releases large amounts of DAMPs and PAMPs, triggering intense immune responses. If uncontrolled, these responses may provoke a cytokine storm, potentially causing severe systemic harm.

PANoptosis involves crosstalk and synergy among multiple signaling pathways, such as the caspase family and RIPKs. During its activation, unintended off-target effects may arise from accidental engagement of pro-survival, proliferative, or anti-apoptotic pathways. Current inhibitors (e.g., necrostatin-1) lack selectivity and may disrupt homeostasis in non-target tissues. For instance, the NLRP12-PANoptosome, activated by heme and PAMPs, promotes acute kidney injury, highlighting challenges in isolating therapeutic benefits from adverse outcomes (32).

In cancer therapy, activating PANoptosis requires careful balancing of its potential safety risks and off-target effects to ensure both efficacy and patient safety.

## 6 Challenges and future directions

PANoptosis, a newly discovered type of programmed cell death, has attracted a lot of attention in cancer research in recent years. As a novel type of programmed cell death, PANoptosis involves various forms of cell death, including pyroptosis, apoptosis, and necroptosis, which interfere with and regulate each other. Although PANoptosis has shown potential applications in cancer study, it still faces many challenges. For example, the molecular mechanisms of PANoptosis are not yet fully elucidated, and whether there are more PANoptosome needs to be further investigated in future studies. The role of small molecule compounds targeting important targets in the PANoptosis pathway in cancer therapy needs to be further explored. In addition, the efficacy and safety of these small molecule compounds need to be verified in clinical trials. It is also necessary to further develop new therapeutic methods such as nanoinducers and virus-mediated gene therapy. Finally, the combination of PANoptosis and other immunotherapy strategies to improve the efficacy of tumor therapy needs further research.

In conclusion, PANoptosis, as a novel cell death mode, plays an important role in the tumor. With the development of research and technology, PANoptosis is expected to become a new target and strategy for tumor therapy.
